# Physiological Changes in Barley *mlo-11* Powdery Mildew Resistance Conditioned by Tandem Repeat Copy Number

**DOI:** 10.3390/ijms21228769

**Published:** 2020-11-20

**Authors:** Cynthia Ge, Paula Moolhuijzen, Lee Hickey, Elzette Wentzel, Weiwei Deng, Eric G. Dinglasan, Simon R. Ellwood

**Affiliations:** 1 Centre for Crop and Disease Management, School of Molecular and Life Sciences, Curtin University, Bentley, WA 6102, Australia; cynthia.ge@curtin.edu.au (C.G.); paula.moolhuijzen@curtin.edu.au (P.M.); Elzette.Wentzel@curtin.edu.au (E.W.); weiwei.deng@curtin.edu.au (W.D.); 2 Centre Queensland Alliance for Agriculture and Food Innovation, The University of Queensland, St. Lucia, QLD 4069, Australia; l.hickey@uq.edu.au (L.H.); e.dinglasan@uq.edu.au (E.G.D.)

**Keywords:** *Hordeum vulgare*, *Blumeria graminis* f. sp. *hordei*, gene silencing, histone modification, reactive oxygen species (ROS)

## Abstract

Wild barley accessions have evolved broad-spectrum defence against barley powdery mildew through recessive *mlo* mutations. However, the *mlo* defence response is associated with deleterious phenotypes with a cost to yield and fertility, with implications for natural fitness and agricultural productivity. This research elucidates the mechanism behind a novel *mlo* allele, designated *mlo-11*(*cnv2*), which has a milder phenotype compared to standard *mlo-11*. Bisulphite sequencing and histone ChIP-seq analyses using near-isogenic lines showed pronounced repression of the *Mlo* promoter in standard *mlo-11* compared to *mlo-11*(*cnv2*), with repression governed by 24 nt heterochromatic small interfering RNAs. The *mlo-11*(*cnv2*) allele appears to largely reduce the physiological effects of *mlo* while still endorsing a high level of powdery mildew resistance. RNA sequencing showed that this is achieved through only partly restricted expression of *Mlo*, allowing adequate temporal induction of defence genes during infection and expression close to wild-type *Mlo* levels in the absence of infection. The two *mlo-11* alleles showed copy number proportionate oxidase and peroxidase expression levels during infection, but lower amino acid and aromatic compound biosynthesis compared to the null allele *mlo-5*. Examination of highly expressed genes revealed a common WRKY W-box binding motif (consensus ACCCGGGACTAAAGG) and a transcription factor more highly expressed in *mlo-11* resistance. In conclusion, *mlo-11*(*cnv2*) appears to significantly mitigate the trade-off between *mlo* defence and normal gene expression.

## 1. Introduction

Barley is an important cereal crop globally, ranked fourth in terms of total quantity produced (http://www.fao.org/in-action/inpho/crop-compendium/cereals-grains/en/). Powdery mildews are obligate biotrophic fungal diseases which in barley are caused by *Blumeria graminis* f. sp. *hordei* (*Bgh*). The fungus stunts barley growth, reduces yield, and can downgrade grain quality from malting to animal feed. Broad-spectrum resistance to powdery mildew that confers resistance to all races of *Bgh* is determined by recessive mutations of the *Mlo* gene. *Mlo* is an enigmatic and broadly conserved plant gene with *mlo*-governed resistance reported in both monocot and dicot species (reviewed in [1]). In barley, over 30 artificially induced *mlo* alleles are known to exist [2]. The gene is responsive to biotic and abiotic stimuli, is involved in developmental processes, and is a negative regulator of cell death [3,4]. *mlo* alleles act via rapid formation of large cell-wall appositions (CWA) and necrosis that correlate with resistance in epidermal cells. The CWA phenotype is also observed in nonhost resistance to powdery mildew, for example, following inoculation of barley with wheat powdery mildew [5].

**Figure 1:**
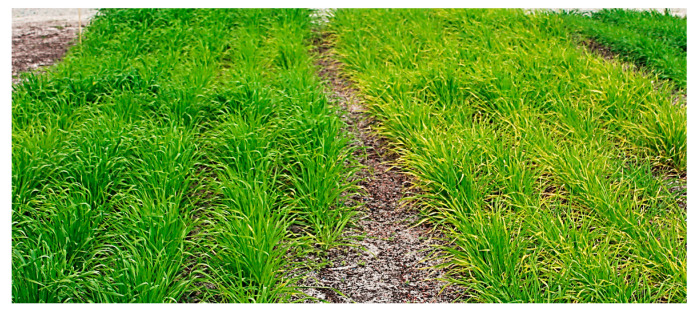
Field-grown barley Pallas 22 (*mlo-5*) plants displaying chlorosis and senescence on the right-hand side, compared to a wild-type *Mlo* line Pallas 18 on the left-hand side.

A natural form of barley *mlo* resistance, known as *mlo-11*, involves a wild-type *Mlo* gene that is silenced via an upstream tandem repeat array consisting of the *Mlo* 5′ UTR and exons 1–5 [6]. Both induced mutants and the original or standard *mlo-11* exhibit undesirable side effects, with necrotic leaf spotting, chlorosis, and leaf senescence (Figure 1). Breeders have overcome these effects, with most European spring cultivars now containing *mlo* resistance [7,8]. Ge et al. [9] uncovered a second natural variant of *mlo-11* with an apparently fewer pleiotropic effects, named *mlo-11*(*cnv2*), which showed partial resistance to powdery mildew in seedling leaves and full resistance in adult leaves. The variant had just two copies of the tandem repeat array, compared to 11–12 in standard *mlo-11*, and lacked spontaneous cell death in uninfected leaves and death of underlying mesophyll cells during infection, characteristic of *mlo* alleles with a stronger effect. Other partial *mlo* resistance phenotypes, conferred by missense mutations, have been reported in barley (*mlo-12* and *mlo-28* [3]) and a series of alleles provide varying levels of potency in wheat [10].

The *mlo-11*(*cnv2*) phenotype appears to correspond to the relative *Mlo* expression level differences compared to standard *mlo-11* [9], and, on the basis of DNA methylation sequencing, the authors postulated that this may involve small RNA-directed DNA methylation (RdDM). DNA methylation was first discovered in plants, and research has consequently demonstrated an elaborate role in plant development and environmental responses through histone modifications [11,12]. Previous studies revealed silencing of transposable elements and repetitive heterochromatic sequences by RdDM [13] and histone H3K9 methylation [14], while repression of regions and genes in euchromatic regions occurs via H3K27 methylation [15]. H3K4 methylation, associated with actively transcribed genes, occurs in euchromatic regions. In addition to methylation, acetylation of histones leads to remodelling of condensed chromatin, leading to gene activation [16].

The precise functions and molecular basis of *mlo* resistance remain little understood, although a role in immunity or suppression of defence responses is implicit with the wild-type gene negatively regulating plant defence responses, with similar sets of co-expressed genes demonstrated in both *Arabidopsis* and barley [4,17,18]. Aside from measurable yield and phenotypic effects associated with *mlo* alleles, resource requirements of this resistance and defence against pathogens in general have been a matter of some conjecture. A trade-off between energy for growth and activation of defence responses was considered to be a question of balancing finite resources and the interactions between different signalling pathways [19]. More recent findings suggest that an antagonistic relationship between growth and defence results from incompatible molecular pathways or sharing of signalling components. One such example is the target genes for the HBI1 transcription factor (TF) which differentially regulates incompatible reactive oxygen species (ROS) requirements, controlling both cell growth and resistance to *Pseudomonas syringae* [20].

Phenotypic differences between the two *mlo-11* variants provided a hypothesis that they act in measurably different growth or defence modes irrespective of the presence or absence of pathogens. With this in mind, we generated near-isogenic lines (NILs) where the resistance domains were introgressed into the barley powdery mildew-susceptible cultivar (cv) Baudin. For comparison, two Pallas NILs [21] with and without the *mlo-5* null mutation were included. The principal objectives of this study were to examine the mechanisms involved in epigenetic gene regulation and to explore global gene expression pathways, common regulatory elements, and resource allocation differences between the two *mlo-11* variants that may explain the phenotypic differences.

## 2. Results

Near-isogenic lines containing standard *mlo-11* and *mlo-11*(*cnv2*) were created. This removes different donor parent genetic backgrounds that may affect phenotypic expression. To confirm *mlo-11* subunit number stability during multiple meiotic events, digital PCR (dPCR) indicated that, excluding one copy of *Mlo* from the wild-type *Mlo* gene, the *mlo-11* subunit repeat numbers were 12 for standard *mlo-11*, two for *mlo-11*(*cnv2*), and zero in cv Baudin (wild-type *Mlo*), abbreviated as S12, S2 and S0, respectively. dPCR subunit copy number averages were 12.213 (standard error (SE) = 0.114, *p* < 0.001), 2.162 (SE = 0.121, *p* < 0.001), and 1.123 (SE = 0.026, *p* < 0.001) for S12, S2, and S0, respectively.

### 2.1. Near-Isogenic Line Macroscopic and Cytological Analysis

**Figure 2:**
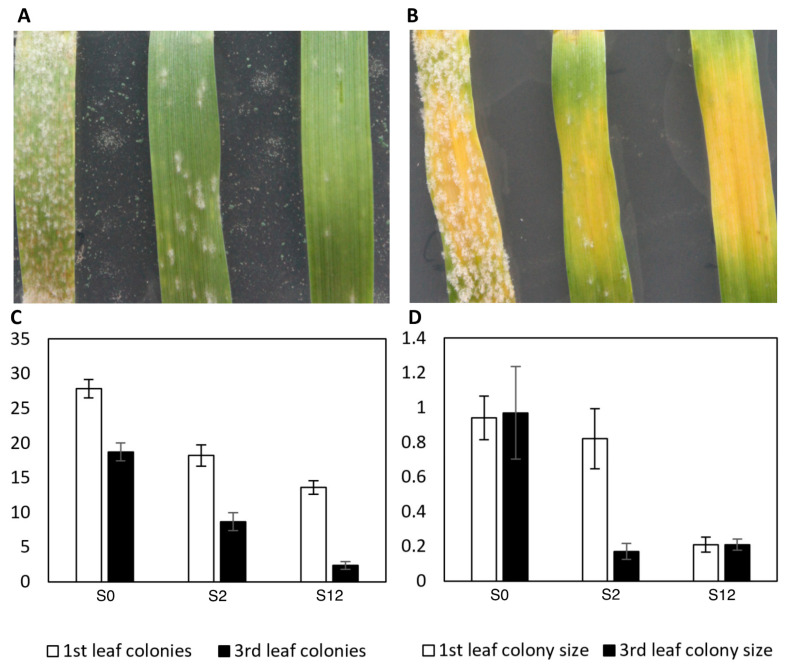
Colony number and size differences in detached leaf assays for the first (**A**) and third leaves (**B**) with isolate Chi-001. Leaves are ordered from left to right by S0 (*Mlo* wild-type control), S2 (*mlo-11*(*cnv2*), and S12 (*mlo-11*), respectively. Colony counts per cm^2^ and sizes in mm are shown in (**C**) and (**D**), respectively, according to five detached leaf replicates per growth stage. Figure 2C shows colony number (*y*-axis) differences between first and third leaves. Figure 2D similarly shows colony size (*y*-axis) differences between first and third leaves. Error bars are standard errors for five replicates (**C**) and (**D**).

Comparative macroscopic and cytological analyses between *mlo-11* variants and wild-type *Mlo* were conducted to establish variant phenotypes in the new NILs. For macroscopic phenotypes, the first- and third-leaf stages were selected to compare the number and size of powdery mildew colonies between the Baudin NILs. The biological significance of these stages lies in the first-leaf stage being generally being more susceptible in *mlo* lines than older leaves, represented by the third leaf. These leaf stages allow differences in onset of *mlo* resistance to be characterised before the emergence of fully expanded fifth leaves, which are coincident with full resistance in milder alleles such as *mlo-11*(*cnv2*) [9]. Detached leaf colony counts showed fewer colonies developed on S2 at the first-leaf stage than on S0 (Figure 2A), while, at the third-leaf stage, colonies progressed more slowly than in S0, with an infection type (IT) 2–3 rather than 4 at 14 days post inoculation (dpi) (Figure 2B). On the third expanded leaves, both S2 and S12 showed much smaller colonies than S0, which had a significantly larger number of colonies than S12 (Figure 2C). On the first expanded leaves, S0 and S2 colony sizes were of similar sizes, whereas S12 were much smaller (Figure 2D). Leaves from the S2 and S12 at the fifth-leaf stage showed no colony formation (data not shown).

**Figure 3:**
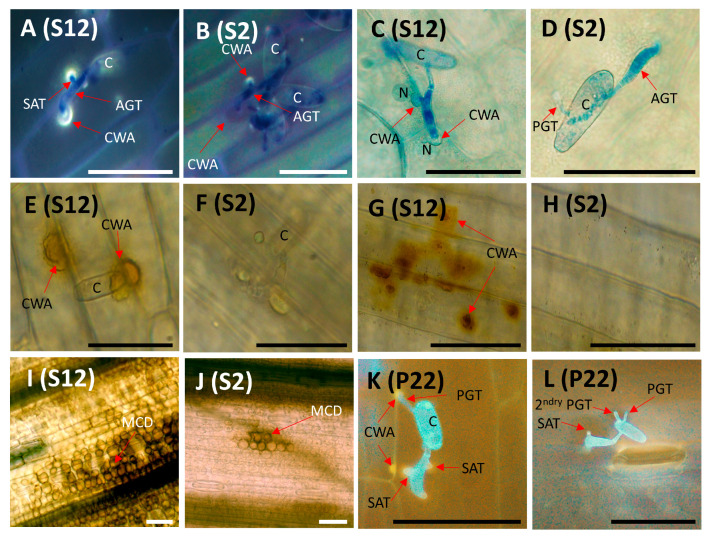
Microscopic images of Baudin near-isogenic line (NIL) responses to attempted penetration by *Blumeria graminis* f sp. *hordei*. (**A**,**C**,**E**,**G**,**I**) are S12 (*mlo-11*). (**B**,**D**,**F**,**H**,**J**) are S2 (*mlo-11*(*cnv2*)). (**K**) and (**L**) are NIL P22 (*mlo-5*). (**G**–**J**) were not inoculated. (**A**,**B**) were double-stained with aniline blue and Evans blue for visualizing cell-wall appositions (CWAs). (**C**,**D**) were stained with trypan blue. (**E**–**J**) were stained with 3,3′-diaminobenzidine (DAB). (**K**,**L**) were triple-stained with aniline blue, calcofluor white, and Evans blue and show *Bgh* phenotypic responses in P22. Abbreviations: AGT, appressorial germ tube; C, conidia; CWA, cell-wall apposition; MCD, mesophyll cell death; N, nucleus; PGT, primary germ tube; SAT, secondary appressorial tube. Scale bar represents 50 µm.

In terms of whole-adult-plant phenotypic differences between the NILs, the most pronounced were for the point mutation *mlo-5* in the Pallas NIL P22 and the control P18. P22 plants grown in outdoor plots showed leaf senescence and physiological spotting compared to their P18 counterparts (Figure 1). At the cytological level, the *mlo* resistance genes in this study shared two prominent features, cell-wall appositions (CWAs or papillae) and mesophyll cell death. Cytological differences occurred in the size and staining intensity of CWA at attempted *Bgh* penetration sites. P22 (*mlo-5*) had the largest and most intensely stained CWA (Figure S1, Supplementary Materials), followed by S12 and S2 (Figure 3A–F). Despite three staining methods being used, (double staining with aniline blue and Evans blue (Figure 3A,B), trypan blue (Figure 3B,C), and 3,3′-diaminobenzidine (DAB) (Figure 3E,F), CWAs were the predominant feature shared by S2 and S12. In the absence of *Bgh* inoculation, S2 lacked spontaneous CWAs or extensive mesophyll cell death (Figure 3H,J), which was present in S12 (Figure 3I) and P22 (not shown), with mesophyll cell death only occasionally observed and limited to a few cells (Figure 3I). Cytosolic vesicles were not obvious in the *mlo* lines at 72 h post infection (hpi). These are considered as multifunctional defence agents and are known to accrue around CWAs at attempted penetration sites by biotrophs [22,23]. We also compared these symptoms with infection by barley by wheat mildew, *B. graminis* f. sp. *tritici* (*Bgt*), where the nonhost interaction at the site of attempted penetration showed weakly defined CWA; however, a large DAB stain halo was evident. CWAs and pigmentation at the penetration site are a feature of biotrophic nonhost interactions [5]. Alongside host cytological responses, the pathogen also displayed different characteristics in different Baudin NIL lines. *Bgh* germinating conidia showed the highest ratio of secondary appressorium tubes (SAT) and secondary primary tubes (PGT) in P22 (Figure 3K,L) and S12 (Figure S1, Supplementary Materials), following unsuccessful primary penetration events.

### 2.2. Small RNA (sRNA) RNA-Directed Mlo Methylation is Associated with Histone Modification

**Figure 4:**
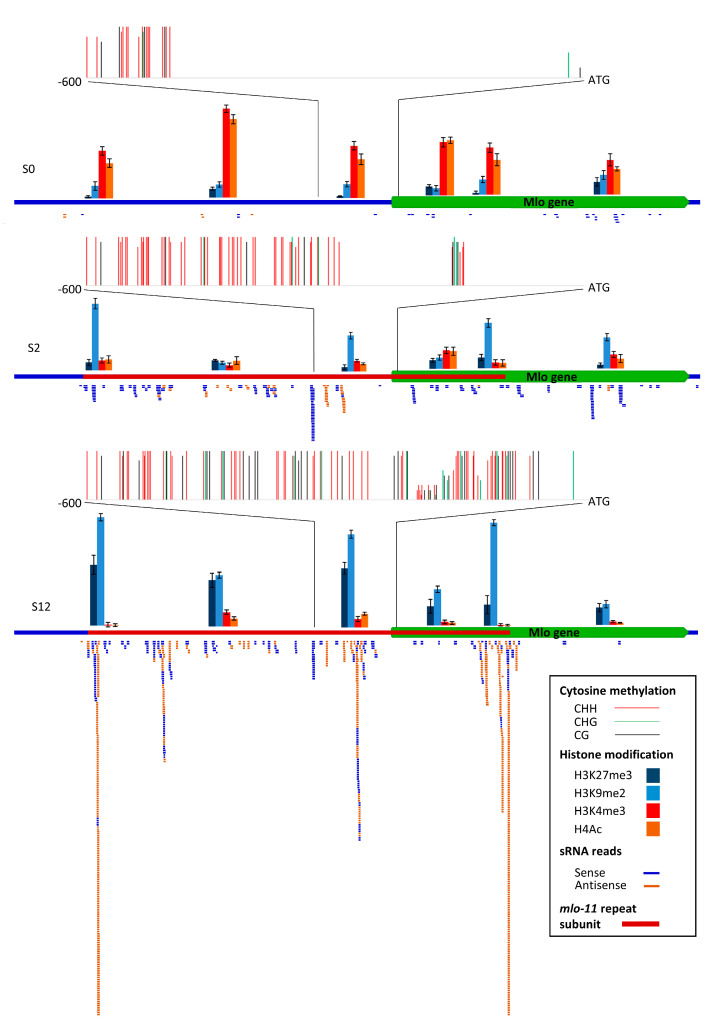
Evidence for small RNA (sRNA)-directed histone modifications at the *mlo-11* subunit between *Mlo* wild type control (S0) and Baudin NILs containing *mlo-11*(*cnv2*) (S2) and standard *mlo-11* (S12). The top panel shows methylation status of a 600 bp region determined by bisulphite sequencing. Red depicts methylation in the CHH context, green the CHG context, and black the CG context (where H = A, T, or C), scaled relative to presence in 10 sequenced replicates. The majority of cytosine methylation is in the CHH context. The middle panel shows levels of histone modifications in six regions determined by chromatin immunoprecipitation (ChIP) PCR. Each histone modification class is scaled is relative to the anti-H3 histone background control. The bottom panel shows sRNA reads mapped across the *mlo-11* repeat subunit.

sRNA sequence alignments to *Mlo* showed that wild-type *Mlo* had very few sRNA reads (an average of 60, Figure 4) which were sense-stranded. For S2, 274 reads of sRNA mapped to the *mlo-11* tandem repeats, as well as *Mlo*. Of these, the largest class was 24 nt (61 reads) with the majority sense-stranded. There is a peak of alignments in a region of antisense small RNA at 300 to 600 bp 5′ to the *Mlo* ATG with a TATA motif, indicative of a TATA box. The sequence within this peak lies at the start of the main block of 5′ untranslated region (UTR) methylation in a CHH context, in addition to an isolated 3′ region shared with S12. Standard *mlo-11* showed significant differences to *mlo-11*(*cnv2*); 1188 reads (880 of 24 nt) sRNA mapped to this variant and, in contrast to *Mlo* and *mlo-11*(*cnv2*), most of the 24 nt sRNA were antisense. The alignment peak region around the TATA was larger than in *mlo-11*(*cnv2*), from around 150 to 600 bp 5′ to ATG, coincident with greater methylation. Standard *mlo-11* also showed substantial alignment antisense peaks that correspond to the start and end of the *mlo-11* subunit repeat.

**Figure 5:**
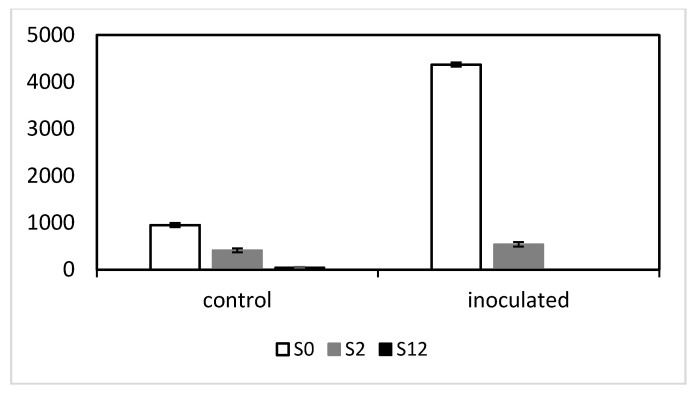
Number of messenger RNA (mRNA) reads mapped to *Mlo* exons 6 to 12 (*Y*-axis) for Baudin NILs containing *mlo-11*(*cnv2*) (S2), *mlo-11* (S12), and wild-type *Mlo* (S0). Suppressed expression for exons 6 to 12 (not present in the *mlo-11* tandem repeats) in S12 is shown, with intermediate suppression in S2, consistent with the epigenetic changes shown in Figure 4. Read counts are averaged across three replicates. The *Y*-axis shows the number of mRNA reads mapped.

Overall, the methylation levels, sRNA antisense read alignments, and histone modifications between the three NILs (Figure 4) support a correlation with *Mlo* expression differences (Figure 5), with the number of sRNA mapped regions higher in S12 than S2, including across the promoter region, and an absence from *Mlo* exons 6–12, which are not present in the *mlo-11* repeat units (Figure 4).

Bisulphite sequencing of a 600 bp region upstream of the *Mlo* start codon showed that, 5′ to the first ~200 bp region next to the *Mlo* ATG, most methylation is in the CHH context (Figure 4). In S12, the first 50 bp and from 240 to 270 bp 5′ to ATG is nonmethylated, and 130–200 bp 5′ to the *Mlo* start codon is partially methylated (around 40%). The remaining sequence is highly cytosine methylated (>90%). For S2 at around 300 to 600 5′ from the start codon, including the position of a peak of aligned antisense sRNA reads, most cytosines are highly methylated.

Since DNA methylation can be linked to both histone modifications and regulation of gene expression [12], we examined modification in six *Mlo* regions. Five of these regions were in the *mlo-11* repeat subunit. These were the repeat start region, 5′ to the promoter region, the promoter region in the vicinity of the TATA box and the 5′ UTR region of the truncated *Mlo* gene, and exon 5 before the repeat truncation site (Figure 4). For a region representing the wild-type *Mlo* gene. we used the *Mlo* exon 9. Chromatin immunoprecipitation (ChIP) followed by qPCR indicated that the key epigenetic markers histone H3 lysine 9 dimethylation (H3K9me2) and H3 Lysine 27 trimethylation (H3K27me3), usually linked to DNA repeat inactivation [24], were high in the promoter region in S12, very low in S0, and intermediate in S2, complementing the bisulphite sequencing. The regions at the start and end of the *mlo-11* repeat subunit showed highest H3K9me2 and H3K27me3 levels in S12, matching antisense sRNA sites, together with a central region (R3). In S2, a similar pattern was evident but with the central region R3 showing highest repression marks. Histone H4 acetylation and H3K4 trimethylation, related to gene activation, showed an inverse correlation with H3K9me2 and H3K27me3, with very low levels in S12 and intermediate levels in S2.

### 2.3. Exon-Specific Expression Differences between the mlo NILs

The colony count and IT results positively correlated with wild-type *Mlo* RNA levels among the five NILs. All RNA reads mapped to *Mlo* are shown in Figure S2 (Supplementary Materials). However, most of the reads for S2 and S12 only mapped to *Mlo* exons 1–5 contained in the *mlo-11* tandem repeat, as well as the 3000 bp promoter region before ATG, with the reads truly determined as wild-type *Mlo* only mapping to *Mlo* exons 6–12 (Figure 5). For P22, since the start codon ATG is point mutated [17], transcription occurs and no functional MLO protein is formed. In S12, very low levels of *Mlo* were evident following inoculation and in noninoculated controls. In the absence of pathogen, wild-type *Mlo* reads in S2 were about half the levels seen in S0. Following infection, *Mlo* in S2 did not increase significantly and was only one-seventh of the level of S0 which increased dramatically, a characteristic reported previously [3].

### 2.4. Whole-Genome Expression Comparisons of the Mlo NILs

**Figure 6:**
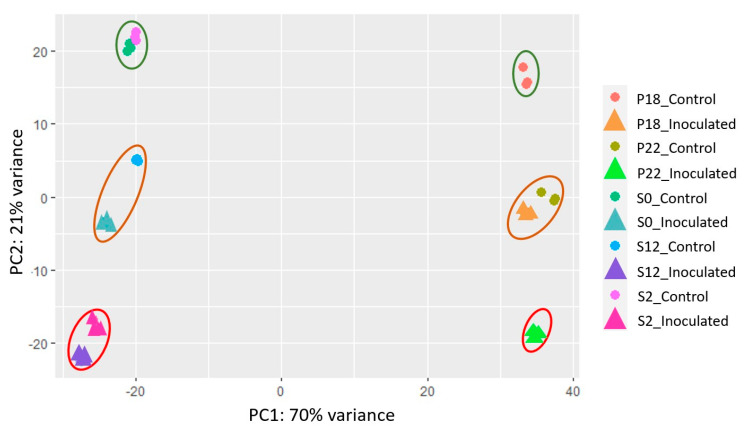
Principal component analysis (PCA) of whole-genome expression for Pallas NILs containing *mlo-5* (P22) and *Mlo* (P18) and Baudin NILs containing *mlo-11* (S12), *mlo-11*(*cnv2*) (S2), and *Mlo* (S0) in control and *Bgh* inoculated samples. PC1 represents the main variance (72%) between Baudin and Pallas genotypes, while PC2 shows the variation (20%) between *Mlo* or *mlo* alleles and treatments (control versus *Bgh* inoculated).

A principal component analysis (PCA) of whole-genome expression during *Bgh* infection further resolved phenotypic differences between all the NILS in this study (Figure 6). On PC1 a variance of 70% was explained by the different genetic backgrounds of the two cultivars Baudin and Pallas. On PC2, a variance of 21% represented different alleles and treatments. Uninfected controls for P18, S0, and S2 all grouped together, P22 and S12 uninfected controls clustered with their respective wild-type *Mlo*-infected samples, and infected S2, S12, and P22 samples grouped at the bottom end of PC2. Uninfected S12 and P22 appear to have similar expression patterns to infected but compatible responses in S0 and P18, respectively.

**Figure 7:**
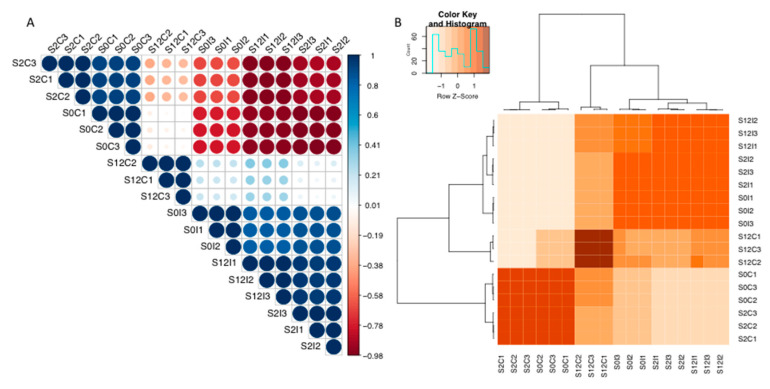
Baudin NILs *mlo-11*(*cnv2*) (S2), *mlo-11* (S12), and *Mlo* wild-type control (S0) sample pairwise correlation coefficient plots of gene expression in *Bgh* inoculated (I) and noninoculated controls (C) for replicates 1–3. (**A**) Positive correlation coefficient (blue) and negative correlation (red) with dots sized by relative correlation *p*-values. (**B**) Heatmap samples are clustered by correlation coefficients. A count histogram is shown in the colour key for values.

Whole-genome expression pairwise correlation coefficients for the Baudin and Pallas NIL samples were analysed (Figure 7 and Figure S3, Supplementary Materials, respectively). The correlation for whole-genome expression between the Baudin NIL replicates showed that S0 and S2 noninfected controls were significantly correlated, but negatively correlated to the S12 control and S0, S2, and S12 infected samples. S2 and S12 infected samples were highly correlated (Figure 7). Whole-genome expression pairwise correlation coefficients and significance for the Pallas NILs showed significant correlations between P18 inoculated and control and P22 control samples, but negative correlations with P22 inoculated samples (Figure S3, Supplementary Materials).

### 2.5. Differential Gene Expression Analysis and Gene Ontology Enrichment between NILs

Among the Baudin NILs, a total of 3470 genes were identified as significantly regulated (Table S1, Supplementary Materials). The Pallas lines exhibited a total of 3312 significantly regulated genes (Table S2, Supplementary Materials). Expression profiles of the Baudin NILs samples (S0, S2, and S12) for the topmost 100 regulated genes showed that a majority of genes were induced in S2 and S12 during infection (Figure S4, Supplementary Materials) compared to the remaining samples. Furthermore, the Baudin NILs clustered into two major groups represented by *Bgh* inoculated and control samples, with a closer grouping for S0 and S2 when noninfected and a closer grouping of S2 and S12 when inoculated by *Bgh* (Figure S4, Supplementary Materials). In the Pallas NILs samples (P22 and P18), the majority of genes were induced in P22 during infection as compared to the remaining samples. Furthermore, the P18 infected grouped with P22 noninfected and noninfected P18 showed the lowest overall expression levels (Figure S5, Supplementary Materials).

**Figure 8:**
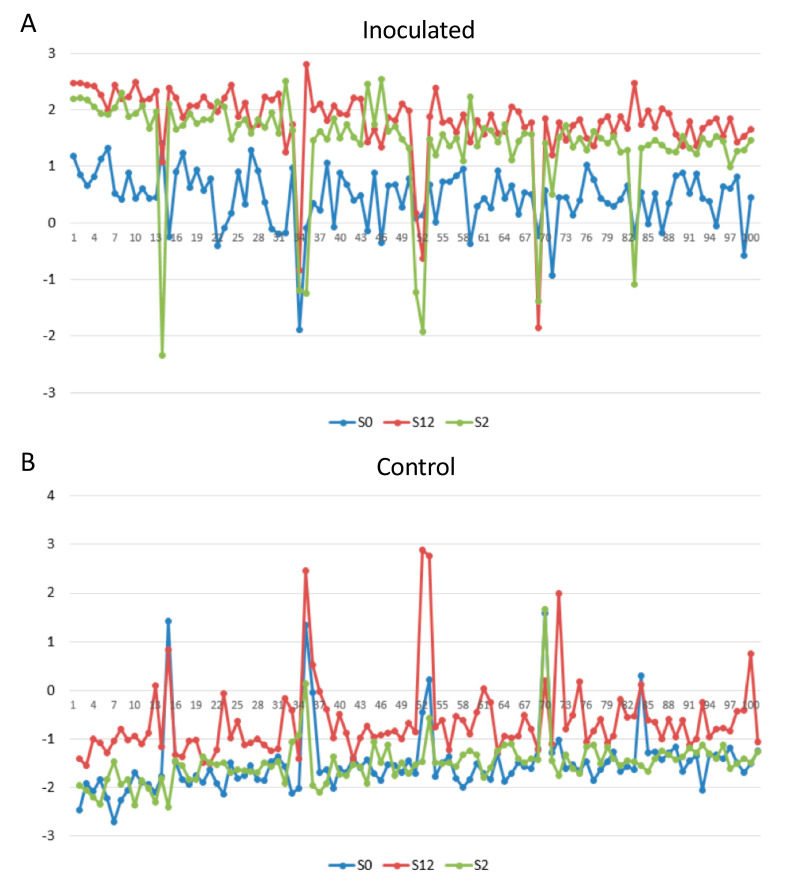
Top 100 differentially expressed genes (*X*-axis) for Baudin NILs containing S12 (*mlo-11*), S2 (*mlo-11*(*cnv2*)), and S0 (*Mlo*) during (**A**) infection by *Bgh* and (**B**) noninoculated control samples, represented through variance-stabilizing transformation (VST) *z*-score (*Y*-axis).

Among the Baudin NILs, expression data for *Bgh* infected samples and noninfected samples (Figure 8) illustrated that S2 oscillates between a wild-type *Mlo*-like resting state when uninfected and S12 levels of expression when infected, and that S12 maintains elevated states of gene expression under both conditions. Six genes notably downregulated for S2 when infected (Figure 8) were represented by three oxidoreductases, one of which (HORVU1Hr1G057860) was downregulated in all three NILs and is annotated as a cytokinin oxidase/dehydrogenase. The remaining two oxidoreductases were downregulated in all three NILs, but to a greater extent in S12 and S2, and are annotated as a monooxygenase (HORVU4Hr1G066230) and a cytokinin oxidase/dehydrogenase (HORVU7Hr1G118130). Two genes specifically downregulated in S2 relative to S12 included a protein involved in catabolism (HORVU3Hr1G006440) and a laccase (HORVU1Hr1G008360). The remaining gene of unknown function (HORVU4Hr1G079440) was downregulated in S2 and to a lesser extent S12, but upregulated in S0.

The top 100 regulated Baudin NIL genes contained 24 biological process Gene Ontology (GO) terms containing 63 genes significantly enriched at a *p*-value ≤ 0.01 (Figure S6, Supplementary Materials). These included response to parasitic fungus (*p*-value = 3.35 × 10^3^), regulation of salicylic acid biosynthesis (*p*-value = 3.35 × 10^3^), and negative regulation of gibberellic acid (*p*-value = 3.30 × 10^−5^). A total of 16 molecular function GO terms were significantly enriched by 62 genes, which included catalytic activity (*p*-value = 8.20 × 10^−5^), hydroperoxide dehydratase (*p*-value = 9.50 × 10^−6^), and calcium ion binding (*p*-value =1.20 × 10^−4^) activities.

The top 100 regulated Pallas genes (Figure S7, Supplementary Materials) contained 54 biological process GO terms containing with 61 genes significantly enriched (*p*-value ≤ 0.01). These included chitin catabolism (*p*-value 4.28 × 10^−5^), protein phosphorylation (*p*-value 3.18 × 10^−7^), and defence response (*p*-value 2.22 × 10^−5^). A total of 39 molecular function GO terms were significantly enriched by 66 genes, which included carbohydrate binding (*p*-value 1.78 × 10^−7^), and protein kinase (*p*-value 2.04 × 10^−8^) and chitinase (*p*-value 2.72 × 10^−5^) activities. Additional differences between *mlo-11* and *mlo-5* NIL GO term biological processes appeared to be those involving innate immune responses and aromatic amino-acid metabolism in *mlo-5*.

### 2.6. A WRKY Transcription Factor Binding Site Is Present in Highly Expressed Genes

The promoter region 1000 bp 5′ from the start codons of the top 100 differentially expressed (DE) genes were extracted and analysed via the MEME software suite [25]. This identified a transcription factor binding motif shared between 16 genes differentially expressed and related to pathogenesis and defence responses. The motif was most similar to the WRKY TF W-box motif (T)TGAC(C/T) [26,27]. Instead of the second thiamine, a guanine is present to give a core sequence GGACT, with an extended consensus of CCGGGACTAA (Figure S8A, Supplementary Materials). Notably, of the top 20 most DE genes, 10 contained this motif within 1000 bp of the start codon (Figure S8B, Supplementary Materials). Gene annotations and their GO terms indicated that these genes are all involved in defence responses. Two of these genes (WRKY33_HORVU1Hr1G070250 and WRKYGK_HORVU1Hr1G080300) were categorised as encoding WRKY TF proteins. A total of 102 barley genes encoding WRKY motifs present in IBSC assembly v2 [28] were investigated and, of those, only the two genes identified here possessed the CCGGGACTAA motif.

### 2.7. Quantitative PCR of Highly Regulated Genes Associated with the New TF Motif

The RNA-seq read count for each of the individual 10 genes associated with an upstream W-box motif were examined and verified by qPCR (Figure S9, Supplementary Materials). The two WRKY-like genes, WRKY33_HORVU1Hr1G070250 and WRKYGQ_HORVU1Hr1G080300, exhibited different expression patterns between inoculated and control samples. Both were most highly expressed in P22 (*mlo-5*) in control samples, particularly WRKYGQ_HORVU1Hr1G080300. However, on infection, all samples showed similar levels of WRKYGQ_HORVU1Hr1G080300 induction, suggesting that the gene is not specifically associated with *mlo* powdery mildew resistance. In contrast, WRKY33_HORVU1Hr1G070250 was most highly expressed in S12 and S2 samples on infection, followed by P22.

The peroxidase HORVU5Hr1G097270 expression increased on infection in proportion with the strength of the *mlo* mutation (P22 followed by S12 and S2). In control samples, peroxidase HORVU5Hr1G097270 was most highly expressed in P22. The oxidase HORVU7Hr1G118130 was repressed in all samples on infection, and most noticeably in S12 and P22, while, in the wild-type *Mlo* lines, it incongruously increased in S0 but not P18. This difference may represent *Bgh* promoting growth in a susceptible line or a genotype-specific response in S0.

HORVU0Hr1G005300 is orthologous to the Downy mildew resistance 6 gene (DMR6, At5g24530) involved in the defence response to oomycetes and is a dioxygenase domain-containing protein. The gene was most highly expressed in infected and control samples of S12. Expression was not associated with the *mlo* mutation or wild-type *Mlo* in the remaining NILs, suggesting that this gene is not involved in *mlo* powdery mildew resistance.

Of the remaining genes, three were pathogenesis-related (PR) such as homologues PR1_HORVU5Hr1G056040, PR1a_HORVU5Hr1G05595 and PRB1-2_HORVU7Hr1G033530, a thaumatin (HORVU5Hr1G051970), and a chitinase (HORVU1Hr1G052430). All were most highly expressed on infection in P22, followed by S12 then S2.

## 3. Discussion

The *mlo-11*(*cnv2*) allele represents an example of modification of *Mlo* gene expression which refines downstream gene expression in a manner that both provides basal defence gene expression levels similar to wild-type *Mlo* in noninfected plants and allows overall expression levels similar to standard *mlo-11* when infected. Similar cytological differences were observed between S2 and S12 variants in the cv Baudin NILs compared to the donor lines described in Ge et al. [9], namely, a lack of spontaneous CWA and mesophyll cell death in S2, together with less prominent CWAs and fewer secondary appressorial and primary growth tubes in seedling leaves. Young S2 NIL plants also showed slightly delayed onset of powdery mildew resistance compared to S12, confirming apparent developmental onset found previously [9,29]. The more obvious CWAs in S12 may be expected to contribute to the earlier complete resistance, although, for effective penetration resistance, CWA polysaccharide composition is considered essential [30].

The sRNA data indicated that antisense 24 nt reads exclusively led to suppressed *Mlo* expression in the *mlo-11* variants. This is consistent with a study by Hackenberg et al. [31], who found that this class of sRNA in barley, in common with other plant species, is mostly associated with repeats. The 24 nt RNA represents heterochromatic small interfering RNAs (siRNAs) [32] which form from double-stranded RNAs derived from a range of sources that produce RNA sense–antisense pairs, including the *mlo-11* subunit tandem repeats in this study. siRNA read alignment data suggested peaks of antisense RNA in the *Mlo* promoter and, therefore, we extended previous bisulphite sequencing of the region [9] to a larger 600 bp section. This showed that antisense RNA alignment sites were highly methylated, indicative of their key role in producing siRNA, and that CHH methylation predominated. In plants, siRNAs are known to be associated with repressive chromatin modifications via 5-methyl cytosine, especially at asymmetric CHH sites [12].

Histone immunoprecipitation followed by qPCR indicated that the repression marks H3K9me2 and H3K27me3 methylation levels were intermediate in S2 compared to S12 and S0, complementing the bisulphite sequencing. Interestingly, in S12 H3K9me2 and H3K27me3, methylation levels were highest in regions at the start and end of the *mlo-11* repeat subunit, together with a central region. S12 appeared devoid of the activation marks H4Ac and H3K4me3, with S2 exhibiting moderate levels although lower than H3K9me2 and H3K27me3. This observation explains the almost complete *Mlo* repression in S12 compared to modest expression in S2 and confirms the inference of Buschges et al. [17] that the *Mlo* phenotype is dependent on the extent of MLO protein inhibition.

Examination of expression profiles for the most highly regulated genes between the Baudin NILs revealed similar gene expression patterns for infected S2 and S12 samples and between S0 and S2 controls. S2 powdery mildew-related gene expression is, therefore, moderated until stimulated by *Bgh* infection. This was also the case for global gene expression by PCA, where the variability between these samples was markedly small, indicating the widespread but conserved effect of *Mlo* inhibition.

Two highly expressed WRKY TFs appear to contain a modified promoter W-box motif, with the consensus sequence CCGGGACTAA rather than (T)TGAC(C/T) [26], although this remains to be experimentally verified. WRKY gene promoters containing W-boxes may act as activators and repressors and interact with a range of proteins, including other TFs, and are extensively involved in auto- and cross-regulation [27]. One of the TFs, WRKYGQ_HORVU1Hr1G080300, was induced to a similar level in all samples. However, WRKY33_HORVU1Hr1G070250 was associated with *mlo* resistance, particularly *mlo-11* resistance. The contribution of this TF to powdery resistance and whether null or alternative alleles naturally occur may have significance in future crop breeding for host resistance.

Pathogen challenge in plants generally causes downregulation of auxins and gibberellins involved in growth in a trade-off with defence responses [19]. The upregulation of salicylic acid biosynthesis and downregulation of gibberellic production were particularly evident in the top 100 most regulated genes for the *mlo-11* NILs compared to *mlo-5*. Other main differences in NIL GO term biological processes between *mlo-11* and *mlo-5* NILs appeared to be those involving innate immune responses and aromatic amino-acid metabolism in *mlo-5*, with the increased aromatic amino-acid metabolism in *mlo-5* plants presumably leading to higher levels of phenolic compounds.

The *mlo* NILs showed greater changes in relative abundance of nicotinamide adenine dinucleotide phosphate (NADPH) oxidases (NOXs) and peroxidases (POXs) during infection. Powdery mildew defence involves reinforcing of the cell wall, while growth, by contrast, often necessitates the loosening of the cell wall to allow cellular expansion. Neuser et al. [20] showed that the TF HBl1 differentially regulates the expression of these two responses through ROS homeostasis within the apoplast (the space between plant cell walls). In growing plants, HBI1 configures apoplastic ROS levels that support growth by activating specific NOX genes and repressing specific POX genes. When challenged by a pathogen, HBI1 is deactivated, reversing the activation of NOX genes and repression of POX genes, resulting in increased apoplastic ROS levels, repressing growth but increasing disease resistance. This is evident in peroxidase_HORVU5Hr1G097270 and oxidase_HORVU7Hr1G118130, where the POX gene is upregulated within the *mlo* resistant NILs upon infection whereas NOX is repressed (Figure S9, Supplementary Materials). ROS are directly antimicrobial and lead to protein cross-linking and CWA formation, while they also have roles in cellular signalling associated with the induction of defence gene expression and the hypersensitive response (reviewed in [33]). The effects of these changes are most extreme in *mlo-5*, followed by S12 then S2. The toxic effects of the ROS and phenolic compound levels in *mlo-5* plants plausibly contribute to their generally senescent phenotype. ROS levels in S12 plants appear to avoid such an extreme effect but still induce spontaneous cell death, while S2 plants show the lowest perturbation.

This study resolves the underlying basis for the *mlo-11*(*cnv2*) phenotype, which is governed by intermediate defence gene expression levels that correlate with the level of antisense 24 nt siRNA mediated *Mlo* suppression. The original landrace containing *mlo-11*(*cnv2*), HOR2543, has no collection information but is thought to have been collected early in the last century alongside much of the ex situ Ethiopian germplasm. The variant allele potentially represents a relatively recent mutational event since the majority of Ethiopian *mlo* mutants are standard *mlo-11*. As a test of natural fitness, the variant’s persistence or selection in new landrace collections may define the evolutionary outcomes of the allele.

## 4. Materials and Methods

### 4.1. Plant Materials

Near-isogenic lines were created at the speed breeding facility at the University of Queensland. Cultivars Westminster (*mlo-11*) and Eth295 (HOR2547, *mlo-11*(*cnv2*)) were backcrossed to a powdery mildew susceptible cultivar containing wild-type *Mlo,* cv Baudin (S0), for five generations (average NIL cv Baudin content equivalent to 96.87%) to give NILs S2 and S12, respectively. PCR primers to identify *mlo-11* progeny during introgression and cleaved amplified polymorphic sequences (CAPS) primers to distinguish Baudin *Mlo* and *mlo-11*(*cnv2*) alleles are presented in Table S3 (Supplementary Materials). Pallas NILs [21] P18 (powdery mildew susceptible, wild-type *Mlo*) and P22 (*mlo-5*) were obtained from Ryan Fowler (Department of Agriculture and Fisheries, Queensland, Australian).

### 4.2. Bgh Culture and Plant Growth Conditions

A monoconidial West Australian *Bgh* isolate, Chi-001, was propagated on detached leaf sections of cv Baudin inserted into 50 mg·L^−1^ benzimidazole agar plates and grown in a Contherm Biosym 6200CP4 incubator (Contherm Scientific Ltd., Petone, New Zealand) at 16 °C and 10 °C under a 12 h light and dark cycle, respectively. Barley plants for cytology, seedling detached leaf infection assays, RNA-seq, and ChIP assays were grown in vermiculite fertilised with Thrive all-purpose soluble plant food (Yates, Clayton, Victoria, Australia) and under light shelves at 300 μmol·m^−2^·s^−1^. Plants for whole-plant infection assays were grown in soil with Nitrophoska Special S (EuroChem Antwerpen NV, Antwerpen, Belgium) in a controlled temperature room (18–22 °C) with a 12 h photoperiod at 450 μmol·m^−2^·s^−1^. A minimum of three replicate plants were used for each accession and treatment, unless specified in the results. Detached leaves and whole plants were inoculated in a settling tower [34] and scored on the 0–4 infection type (IT) scale of Mains et al. [35], where 0 = no visible symptoms, 1 = sparse mycelial development with no sporulation, 2 = mycelial present with very few spore chains, 3 = moderate mycelial development and discrete lesions with sporulation, and 4 = amorphous mycelial development and abundant sporulation.

### 4.3. Cytology and Detached Leaf Inoculations

*Bgh* infected leaves were sampled at 72 h post infection (hpi) by taking 0.5 cm^2^ leaf segments from 10 independent leaves per genotype. Triple staining with Evans blue, aniline blue, and calcofluor white, double staining with staining with Evans blue and aniline blue, and DAB (3,3′-diaminobenzidine) and trypan blue were described previously [9]. Fluorescence for double- and triple-stained samples was detected using the Zeiss filter set 38 (Carl Zeiss Microscopy, White Plains, NY, USA: BP 470/40 nm; FT 495 nm; BP 525/50 nm).

Powdery mildew colony counts and size measurements of individual genotypes were performed with detached leaves using five replicates per growth stage at the first- and third-leaf stages. Leaves were severed, inserted onto benzimidazole agar plates, and inoculated using a powdery mildew settling tower [34]. *Bgh* colonies were counted within 1 cm^2^ leaf sections at 14 days post inoculation. Colony diameters were estimated for 10 colonies per replicate detached leaf.

### 4.4. Whole-Genome RNA and Small RNA Sequencing

For each barley line (S0, S2, S12, P18, and P22), leaf tissue was sampled from three biological replicates at 48 hpi for infected samples together with noninoculated controls. All plants were grown in a high-humidity chamber under the same conditions. Leaf tissue was sampled from each accession at the third-leaf stage. Total RNA was extracted using an RNeasy Plant Mini Kit (Qiagen, Hilden, Germany) following the manufacturer’s protocol. For RNA-seq, RNA libraries were made with an Illumina TruSeq RNA v2 kit and sequenced with 150 bp paired-end reads on an Illumina HiSeq 2500 platform (Illumina Inc., San Diego, CA, USA) at the Novogene Institute (Beijing, China) using stranded protocols. The Illumina CASAVA1.8 pipeline was used to generate sequence data. Small RNA (sRNA) sequencing was performed by the Novogene Institute on an Illumina HiSeq 2500, with read cleaning of sequencing adapters and non-sRNA contaminants. The RNA-seq read data were deposited at DDBJ/ENA/GenBank under accession PRJEB39864 and sRNA-seq data were deposited at DDBJ/ENA/GenBank under accession PRJEB40118.

### 4.5. RNA-seq Analysis

RNA-seq reads were quality checked with FastQC v0.11.5 [36] and trimmed with a Phred score ≥30 using Trimmomatic v0.33 [37] with HEADCROP:12, ILLUMINACLIP:TruSeq-PE.fa:2:30:5, LEADING:10, TRAILING:10, SLIDINGWINDOW:4:25, and MINLEN:50 parameters. Quality trimmed reads were aligned to the International Barley Sequencing Consortium (IBSC) barley genome version 2.43 [28] using the RNA-seq aligner Star version 2.7.0e [38] with quantMode equal to GeneCounts. Gene expression counts were calculated and guided by the barley genome version 2.43 gene annotations. Normalisation, gene dispersion estimates, and differential gene expression were performed using R v3.5.1 package DESeq2 version 1.22.2 [39]. A sample principal component analysis (PCA) was conducted on expression data using variance-stabilizing transformation (VST) across the five lines (Baudin (S0), S2, S12, P18, and P22) for inoculated and noninoculated samples. The most significant differential gene expression between the samples was set at ≥2-fold change and a Benjamini–Hochberg (BH) adjusted *p*-value (false discovery rate) ≤0.05 [40]. Plots were constructed using R v3.5.1 and tidyverse v1.3.0, ggpubr v0.2.4, and ggplot2 v3.2.1 libraries. Pearson’s ranked correlation coefficient analysis was conducted with the R v3.5.1 corrplot v0.84 package and gene expression plots with pheatmap v1.0.12. All markdown R documents and data are available through GitHub (https://github.com/ccdmb/barley-pow-rnaseq).

To estimate *Mlo* exon read counts, the forward-stranded reads that mapped to the barley genome were extracted using SAMtools v1.7 view [41], with filters for SAM flags, second in pair (−f 128), and then the reverse reads filtered for read reverse strand (0 × 10) first in pair (0 × 40) (−f 80) before being merged. Forward-stranded *Mlo* exons with 6–12 reads were counted for each sample using BEDTools v2.26.0 [42]. Small RNA-seq data was mapped against the barley *Mlo* region after normalising to 21 m reads per sample using the Map to Reference function in Geneious v. R8 [43].

### 4.6. Identification of Transcription Factor Binding Sites of Highly Regulated Genes and qPCR

One kilobase sequence regions upstream of the ATG start codon for all highly regulated genes were extracted using EnsemblPlants Biomart [28] and submitted to MEME Suite 5.1.1 [44], TOMTOM, and JASPAR CORE 2018 to identify transcription factor binding sites. Quantitative RT-PCR for the 10 most highly regulated genes containing a WRKY motif of interest was performed on a CFX96 Touch Real-Time PCR Detection System (Bio-Rad, Hercules, CA, USA) with SYBR green and Platinum DNA polymerase (Invitrogen). The barley actin gene was used as an internal reference, and relative transcript levels of biosynthesis were calculated with the ΔΔCt method, factoring in primer amplification efficiencies. qPCR primers used are described in Table S3 (Supplementary Materials). Student’s *t*-tests between and control and inoculated were calculated for each NIL using three biological replicates with a significance value less than 0.001.

### 4.7. Gene Ontology Term Enrichment of Significantly Differentially Expressed Genes

The top 100 genes with the largest expression variance across the five lines (Baudin (S0), S2, S12, P18, and P22) for inoculated and noninoculated samples were selected for Gene Ontology analysis. Singular enrichment analysis (SEA) of the top 100 genes was performed using agriGO [45]. The agriGO SEA selected parameter settings used were for a hypergeometric statistical test with the Yekutieli (false discovery rate under dependency) multi-testing adjustment method, significance level ≤ 0.05, five minimum mapping entries, and Gene Ontology type Plant GO Slim [46,47]. The top 100 genes from a variance-stabilizing transformation (VST) for Baudin and Pallas NILs were also individually analysed, together with an enrichment analysis for GO terms using R topGO v2.34.0 with a classic Fisher test and significance level ≤0.05.

### 4.8. Chromatin Immunoprecipitation

ChIP assays were performed as previously described [48,49] with three biological replicates and three technical replicates. Antibodies used in ChIP assays included anti-H4Ac 06-866 (MilliporeSigma, St. Louis, MO, United States) anti-H3K4me3 (ab8580, Abcam PLC, Cambridge, UK), anti-H3K27me3 (Abcam, ab6002), and anti-H3K9me2 (Abcam, ab1220), and polyclonal anti-H3 histone background control (Abcam, ab1791). The amount of immunoprecipitated chromatin was determined by qPCR in different *Mlo* (R1-R6) regions using specific primers (Table S3, Supplementary Materials). Relative abundance of chromatin was normalised to the amount of DNA immunoprecipitated by a histone 3-specific antibody.

### 4.9. Digital PCR and Bisulphite Sequencing

Digital PCR was performed to determine the stability of the *mlo-11* subunits in NILs S2 and S12 following backcrossing. Baudin (S0) was used as the wild-type control, using the protocol described in Ge et al. [9] and the primers in Table S3 (Supplementary Materials).

The EZ DNA Methylation-Direct kit (Zymo Research, Irvine, CA, USA) was used to perform bisulphite treatment of genomic DNA. First, 200 ng of each accession with three replicates of *Bgh*-infected and noninfected samples was subjected to cytosine–thiamine conversion. The converted DNA was amplified by hot-start PCR with Takara Ex Taq (Clontech Takara Cellartis, Shiga, Japan). Untreated DNA was used in control reactions. Amplified fragments were cloned into the pGEM-T vector (Promega, Madison, WI) and 18 ligated clones from each accession were Sanger sequenced.

## Supplementary Materials

Supplementary materials can be found at https://www.mdpi.com/1422-0067/21/22/8769/s1.
