# Supplementary material for: Physiological Changes in Barley mlo-11 Powdery Mildew Resistance Conditioned by Tandem Repeat Copy Number
Source: Int J Mol Sci. 2020 Nov 20;21(22):8769. doi: 10.3390/ijms21228769 (PMC7699567; doi:10.3390/ijms21228769)

Physiological changes in barley *mlo-11* powdery mildew resistance conditioned by tandem repeat copy number

Cynthia Ge[1], Paula Moolhuijzen[1], Lee Hickey[2], Elzette Wentzel[1], Weiwei Deng[1], Eric G. Dinglasan[2] and Simon R. Ellwood[1*]

[1] Centre for Crop and Disease Management, School of Molecular and Life Sciences, Curtin University, Bentley, WA 6102, Australia
[2] Centre Queensland Alliance for Agriculture and Food Innovation, The University of Queensland, St Lucia, Australia
* Correspondence: simon.ellwood@curtin.edu.au

Supplementary Figure 1. Phenotypes for host CWA and pathogen responses in barley seedling leaves of NILs containing *mlo-5* (P22), *mlo-11*(*cnv2*) (S2) and *mlo-11* (S12) alleles inoculated with *Blumeria graminis* f. sp. *hordei*. Control lines with wild-type *Mlo* are Pallas 18 (P18) and cv Baudin (S0). Non-host response results against *Blumeria graminis* f. sp. *tritici* (*Bgt*) are shown for cv Baudin. Standard error values are for ten geminating conidia in three replicate leaves. Individual lines were determined to be significantly different using a Student's t-test at $P < 0.001$.

	First appressorium CWA DAB colour intensity : diameter (µm) *P*<0.001	Secondary appressorium tube ratio *P*<0.001 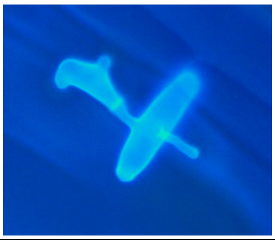	Secondary primary tube ratio *P*<0.001 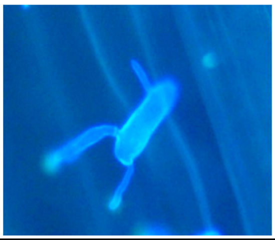
P22	Dark brown DAB CWA staining 3.71 (s.e. 0.0723)	0.92 (s.e. 0.0274)	0.64 (s.e. 0.03247)
S12	Medium brown DAB CWA staining : 3.89 (s.e. 0.0926)	0.87 (s.e. 0.0379)	0.43 (s.e. 0.02980)
S2	Light brown DAB CWA staining : 3.12 (s.e. 0.0977)	0.62 (s.e. 0.03762)	0.37 (s.e. 0.04728)
P18	Less developed CWA with no DAB staining : 1.54 (s.e. 0.0623)	0.18 (s.e. 0.04152)	0.07 (s.e. 0.03725)
S0	Less developed CWA with no DAB staining : 2.03 (s.e. 0.0872)	0.16 (s.e. 0.02754)	0.07 (s.e. 0.02538)
S0 inoculated with *Bgt*	Dark brown DAB CWA staining together with a large DAB halo : 8.29 (s.e. 0.1291)	0.03 (s.e. 0.01342)	0.87 (s.e. 0.07593)

Supplementary Figure 2. Total *Mlo* RNA-seq read counts for barley NILs containing *mlo-5* (P22), *mlo-11* (S12), and *mlo-11*(*cnv2*) (S2). Control lines with wild-type *Mlo* are Pallas (P18) and Baudin (S0). Results are shown for *Blumeria graminis* f. sp. *hordei* inoculated and non-inoculated samples with three replicates.

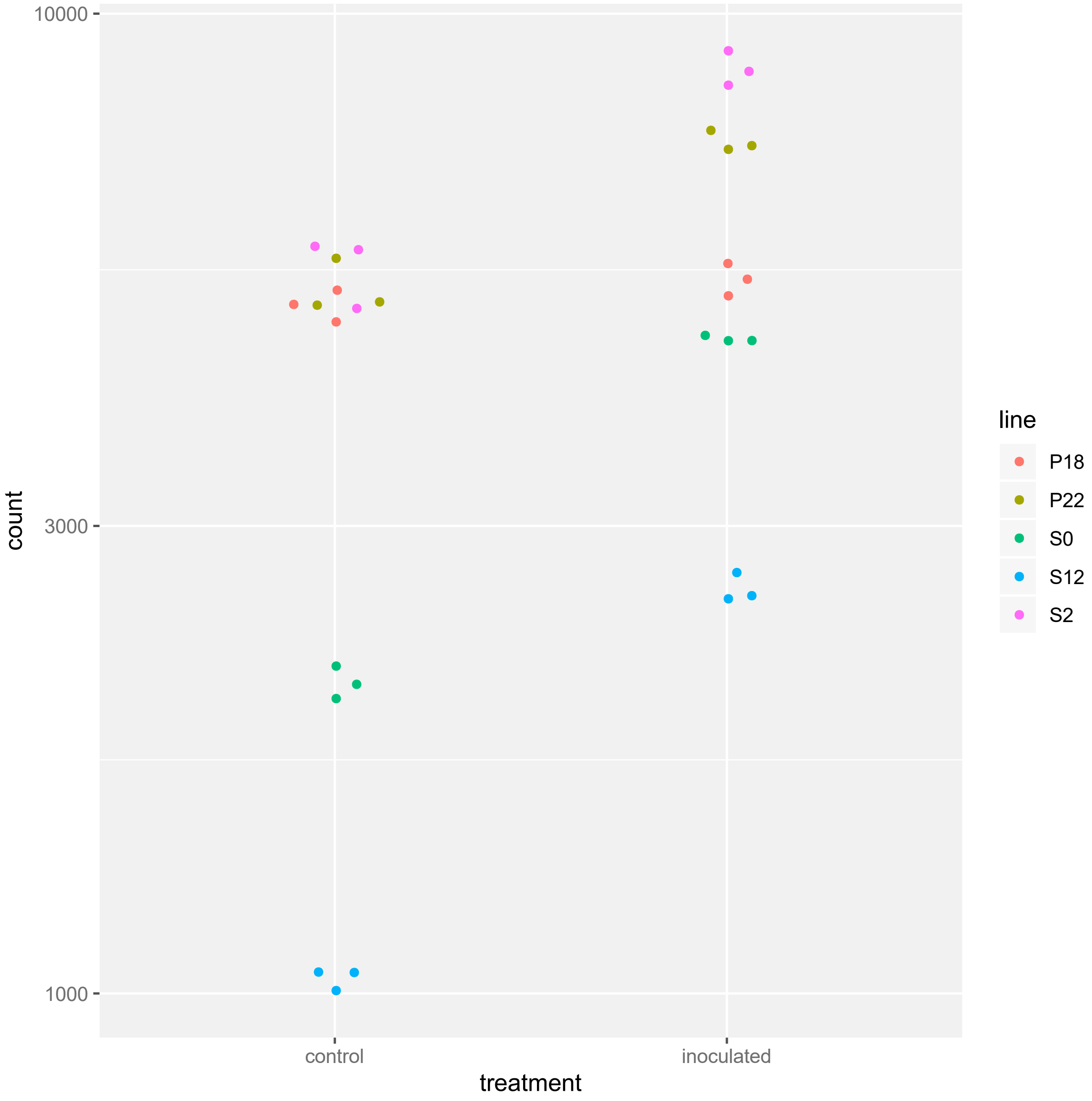

Supplementary Figure 3. Pallas NIL for *mlo-5* (P18) and *Mlo* wild-type control (P22) sample pairwise correlation coefficient plots of gene expression in *Blumeria graminis* f. sp. *hordei* inoculated (I) and non-inoculated controls (C) for replicates 1-3. A) Sample positive correlations (blue) and negative correlations (red) are shown with circles sized by the relative correlation *P*-values. B) Heat map samples are clustered by correlation coefficients, showing that P22 inoculated samples are more distant to all other samples. A count histogram is shown in the colour key for values.

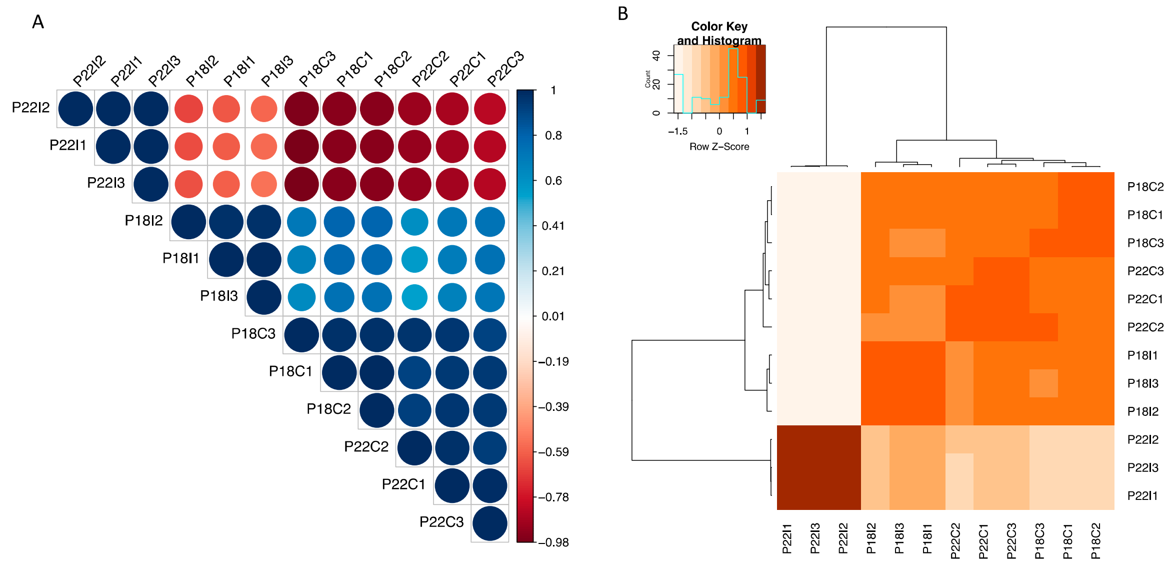

Supplementary Figure 4. Baudin NIL gene expression (expression - mean expression) heatmap of the top 100 most highly regulated genes. Data is presented for *mlo-11*(*cnv2*) (S2), *mlo-11* (S12), and *Mlo* wild-type control (S0) alleles, for *Blumeria graminis* f. sp. *hordei* inoculated and non-inoculated control samples. Hierarchical clustering of genes (vertical tree for rows) shows the majority of genes are induced in S2 and S12 inoculated as compared to the S0 inoculated and control treatments. Hierarchical clustering of samples (horizontal tree for columns) shows agreement for sample replicate gene expression and the clustering of samples into two groups related to treatments.

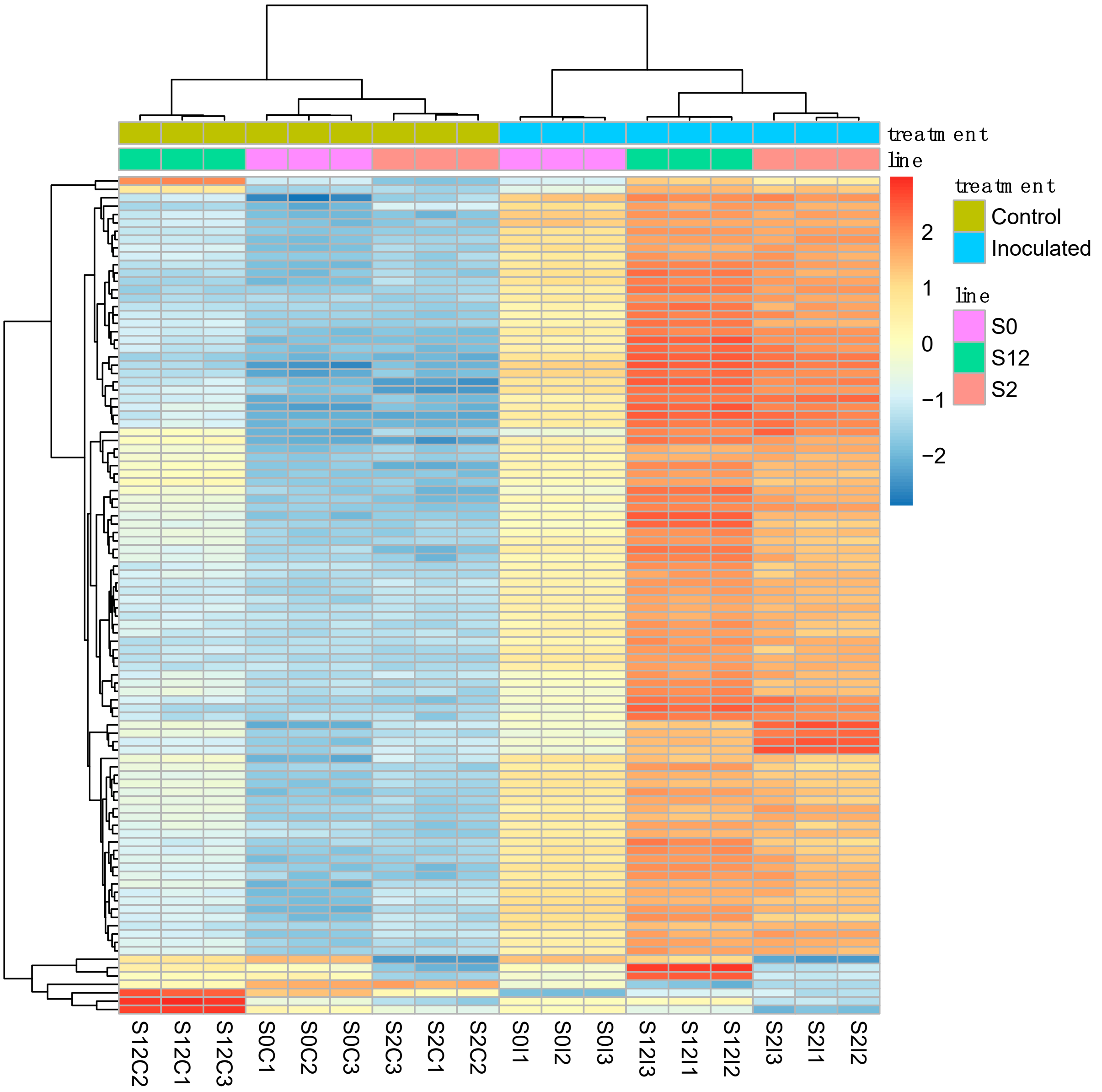

Supplementary Figure 5. Pallas NIL gene expression (expression - mean expression) heatmap of the top 100 most highly regulated genes. Data is presented for *mlo-5* (P18) and *Mlo* wild-type control (P22) alleles, for *Blumeria graminis* f. sp. *hordei* inoculated and non-inoculated control samples. Hierarchical clustering of genes (vertical tree for rows) shows that most of top 100 genes are induced in P22 infected as compared to the P18 infected and control treatments. Hierarchical clustering of samples (horizontal tree for columns) show agreement between sample replicate gene expression and the clustering of samples into two groups, consisting of P22 inoculated and the remaining samples.

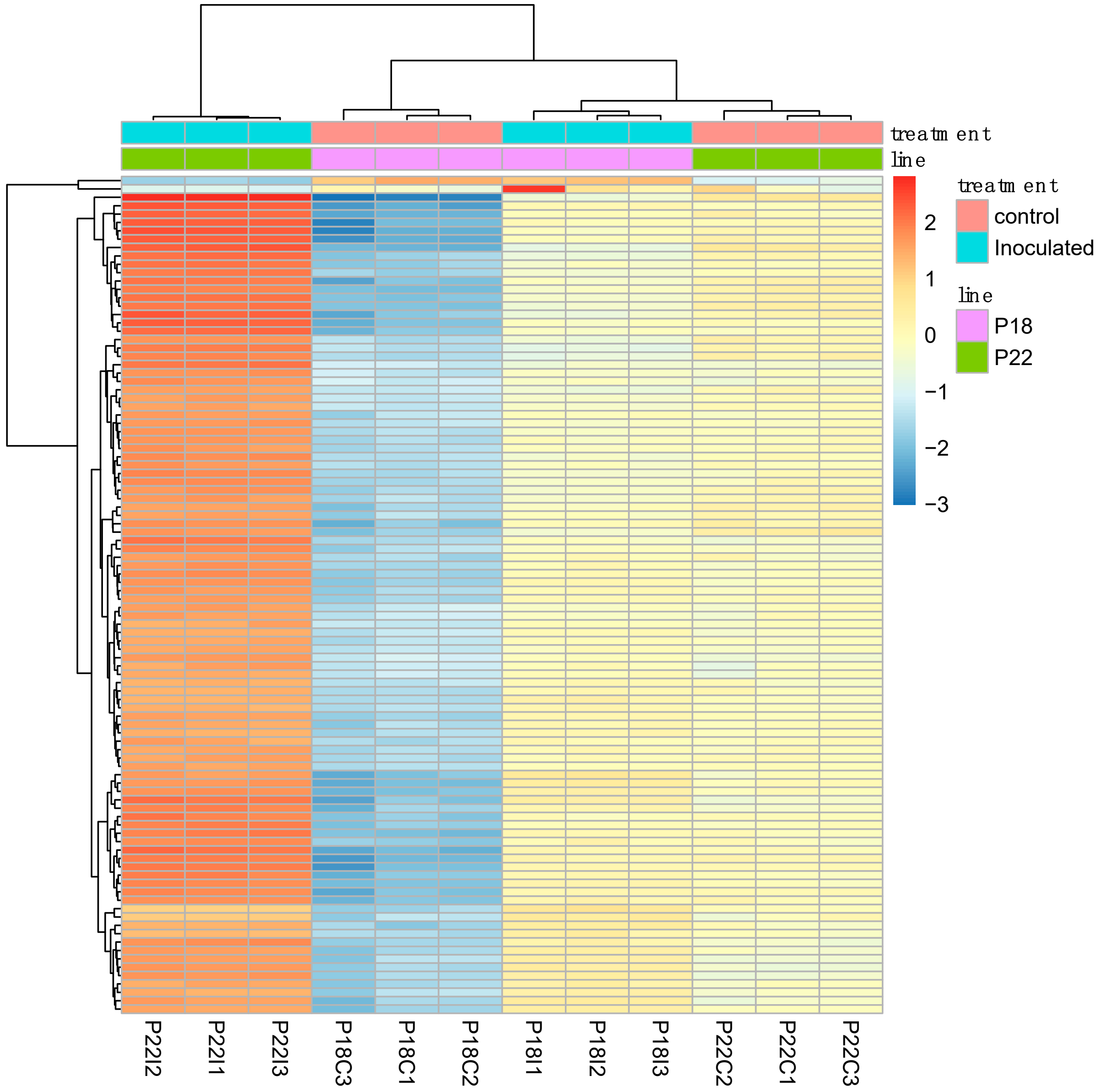

Supplementary Figure 6. Bar charts for significantly enriched GO terms based on NILs for *mlo-11*(*cnv2*) (S2), *mlo-11* (S12), and a *Mlo* wild-type control (S0), across all samples inoculated or non-inoculated with *Blumeria graminis* f. sp. *hordei*. Bar charts represent the 100 most highly regulated genes. A) GO terms for molecular functions. B) GO terms for biological processes.

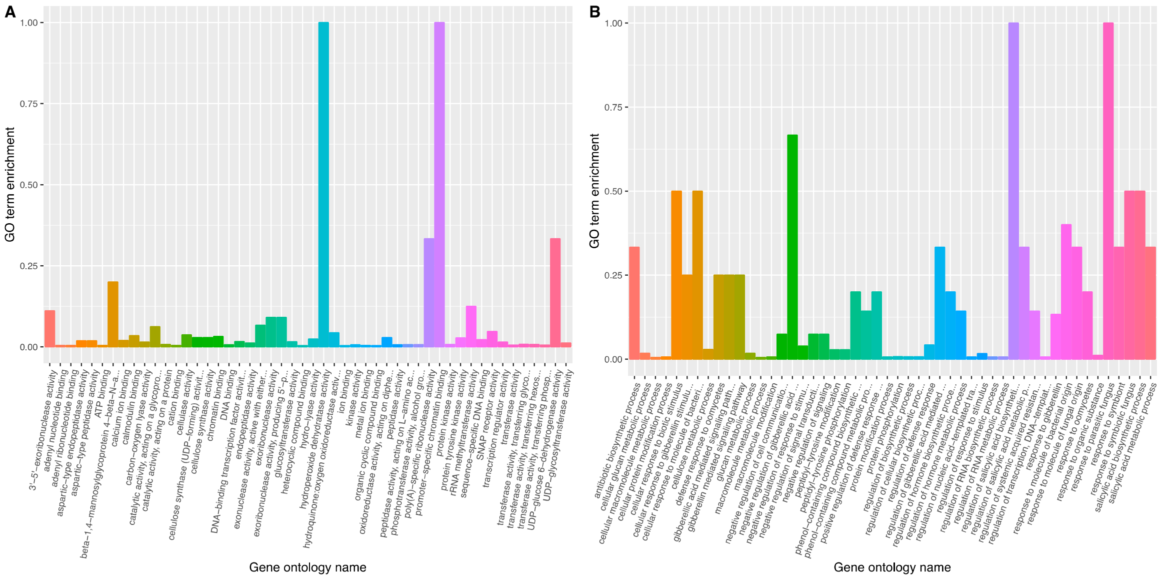

Supplementary Figure 7. Bar charts for significantly enriched GO terms based on a NILs for *mlo-5* (P18) and a *Mlo* wild-type control (P22), across all samples inoculated or non-inoculated with *Blumeria graminis* f. sp. *hordei*. Bar charts represent the 100 most highly regulated genes. A) GO terms for molecular functions. B) GO terms for biological processes.

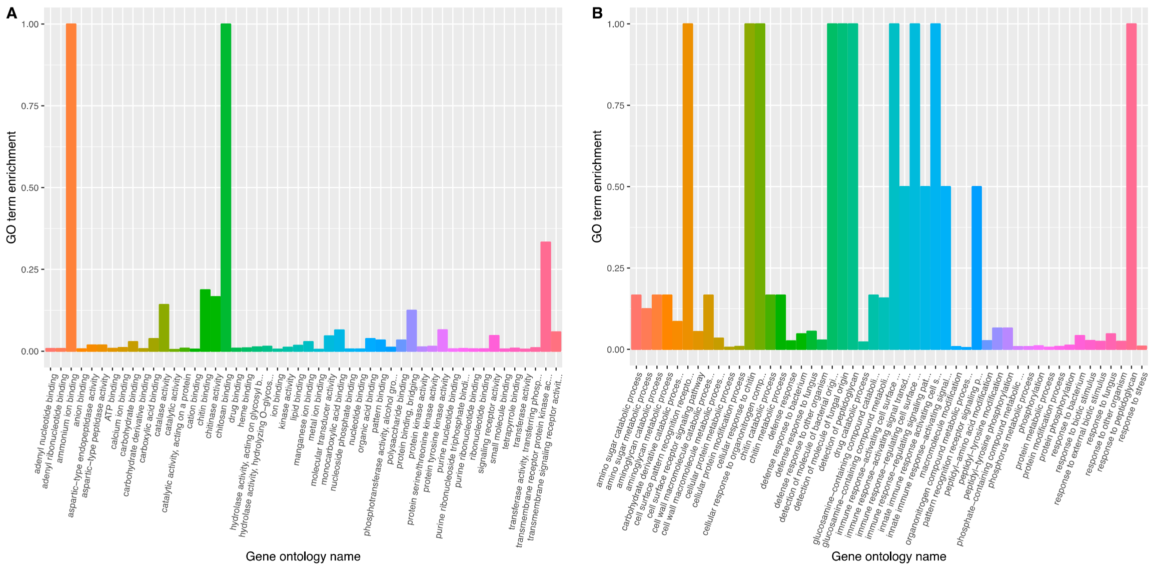

Supplementary Figure 8. Ten of the first 20 top differentially expressed genes among NILs P22 (*mlo-5*), P18 (*Mlo*), S12 (*mlo-11*), S2 (*mlo-11*(*cnv2*)) and S0 (*Mlo*) showed a new consensus WRKY motif (**A**). **B**, the motif position in a 1000 bp region upstream of the *Mlo* start codon ATG of the 10 topmost differentially expressed genes. All ten genes are involved with plant defence responses.

A

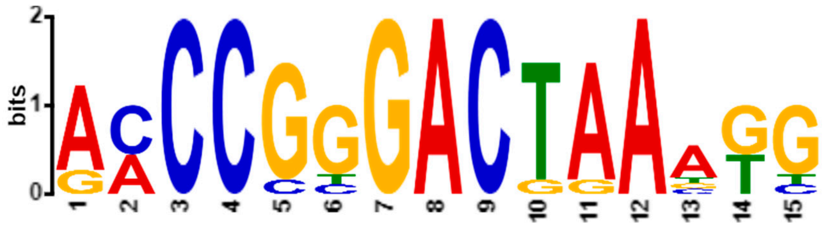

B

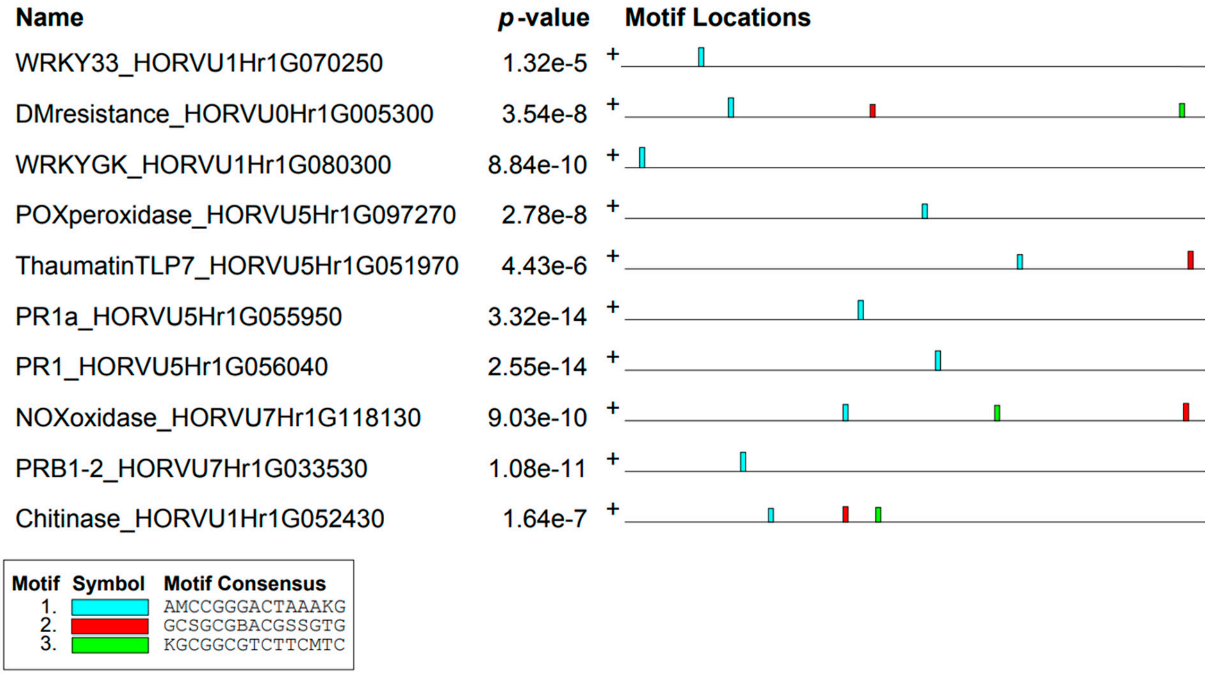

Supplementary Figure 9. RNA-seq raw read count (A) and real-time qPCR expression (B) for 10 most upregulated genes associated with an upstream W-box motif binding site among all barley NILs for *Blumeria graminis* f. sp. *hordei* inoculated and control samples. Three replicates were used for each NIL; P22 (*mlo-5*), P18 (*Mlo*), S12 (*mlo-11*), S2 (*mlo-11*(*cnv2*)) and S0 (*Mlo*). The fold change relative to barley actin is shown on the y-axis in the in real-time qPCR plots. Students t-tests between and control and inoculated were calculated for each barley line. Comparisons not significant at a *P*-value < 0.001 are indicated by "#" and significant at a *P*-value < 0.05 indicated by "&" on the real-time qPCR x-axis.

WRKY33_HORVU1Hr1G070250:

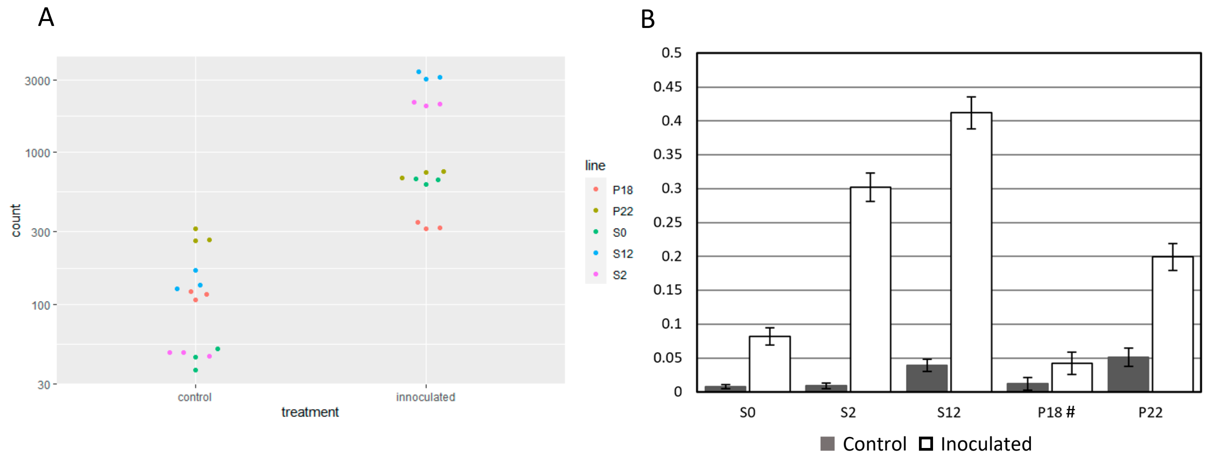

Downy_Mildew_Resistance_HORVU0Hr1G005300:

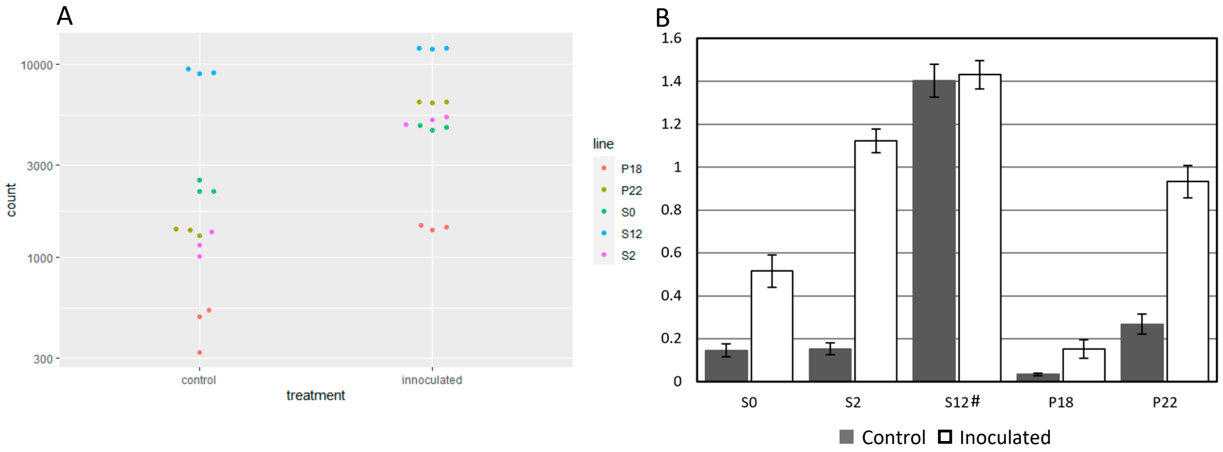

WRKYGQ_HORVU1Hr1G080300:

A

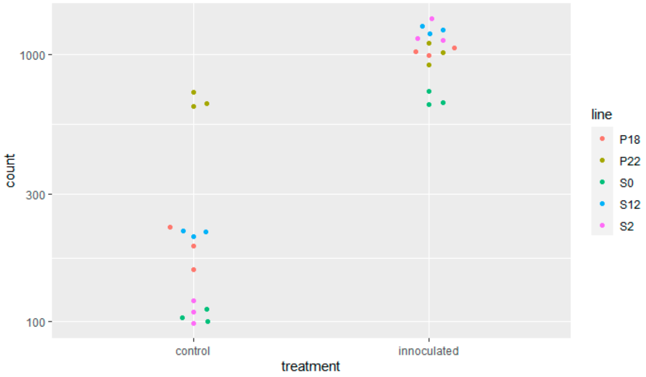

B

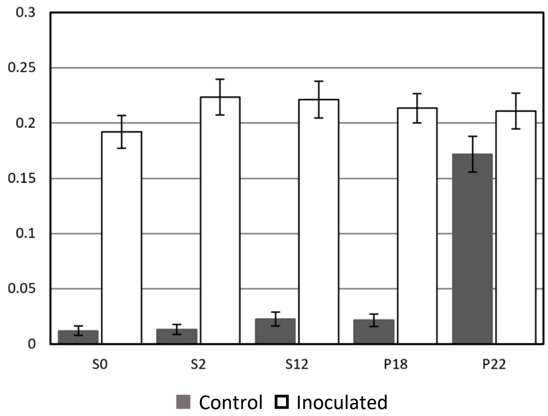

POXperoxidase_HORVU5Hr1G097270:

A

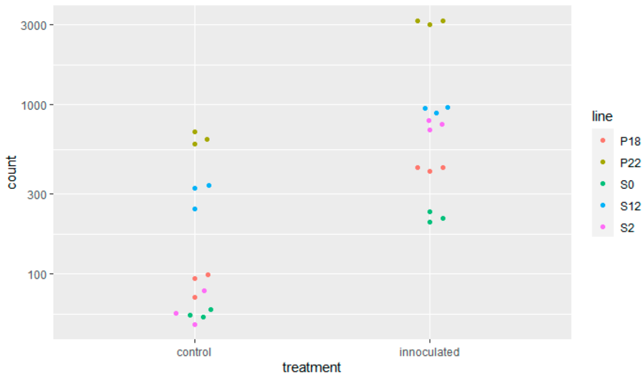

B

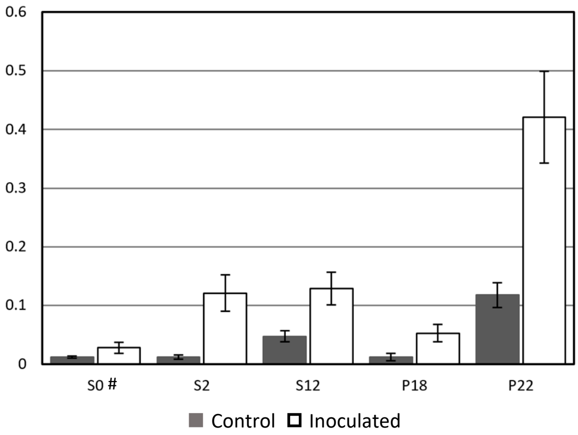

ThaumatinTLP7_HORVU5Hr1G051970:

A

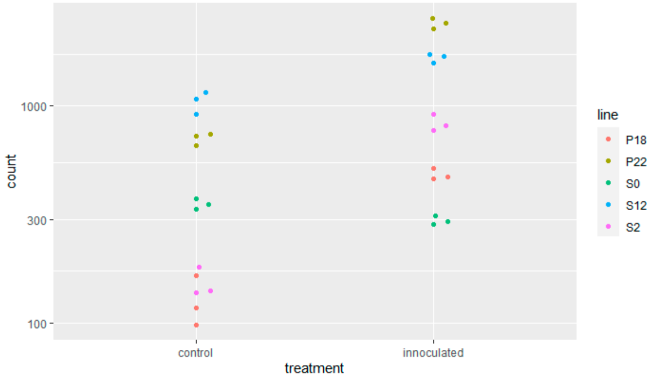

B

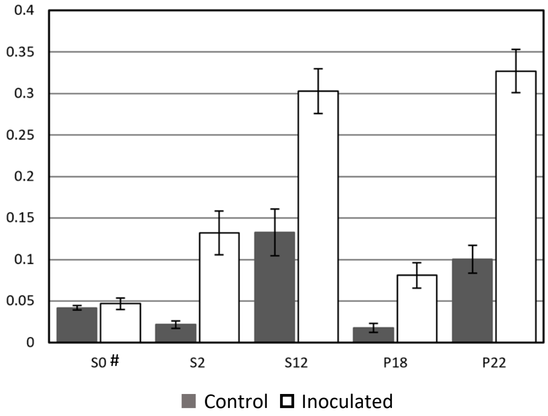

Pathogenesis-related _protein_1a_HORVU5Hr1G055950 (PR1a_HORVU5Hr1G055950):

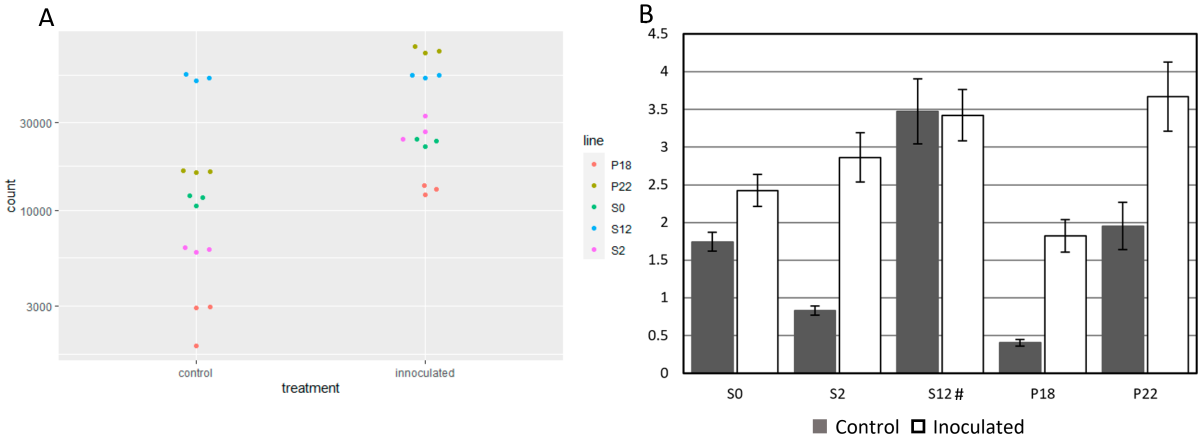

Pathogenesis-related _protein_1_HORVU5Hr1G056040 (PR1_HORVU5Hr1G056040):

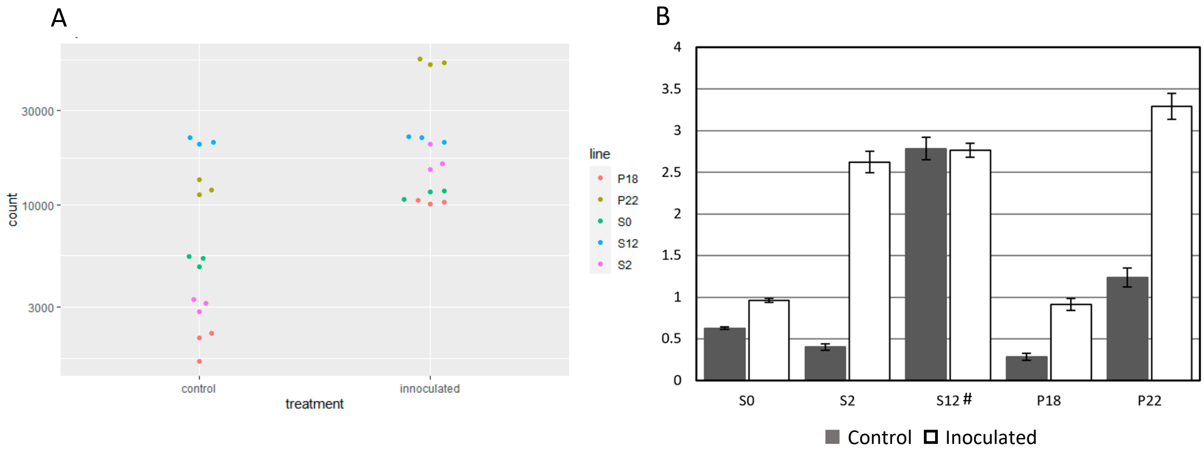

NOXoxidase_HORVU7Hr1G118130:

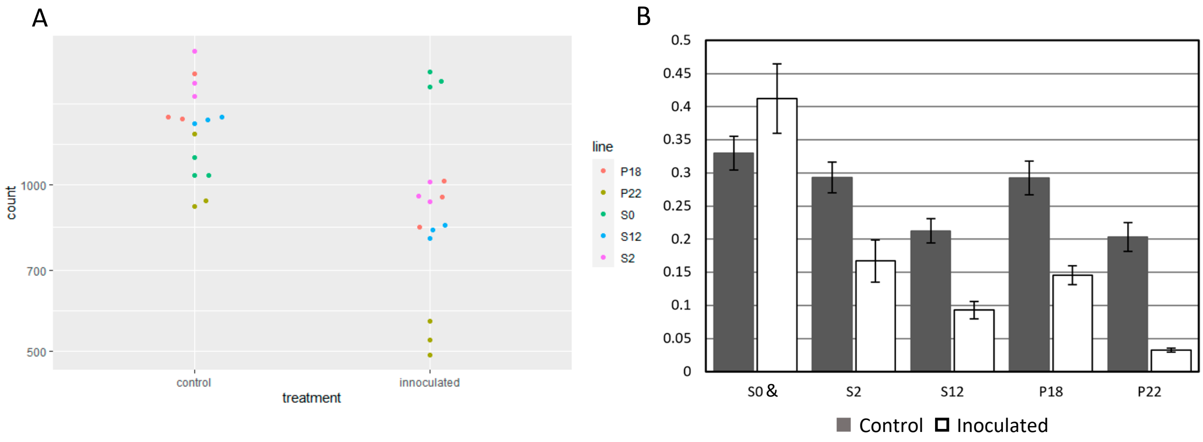

Chitinase_HORVU1Hr1G052430:

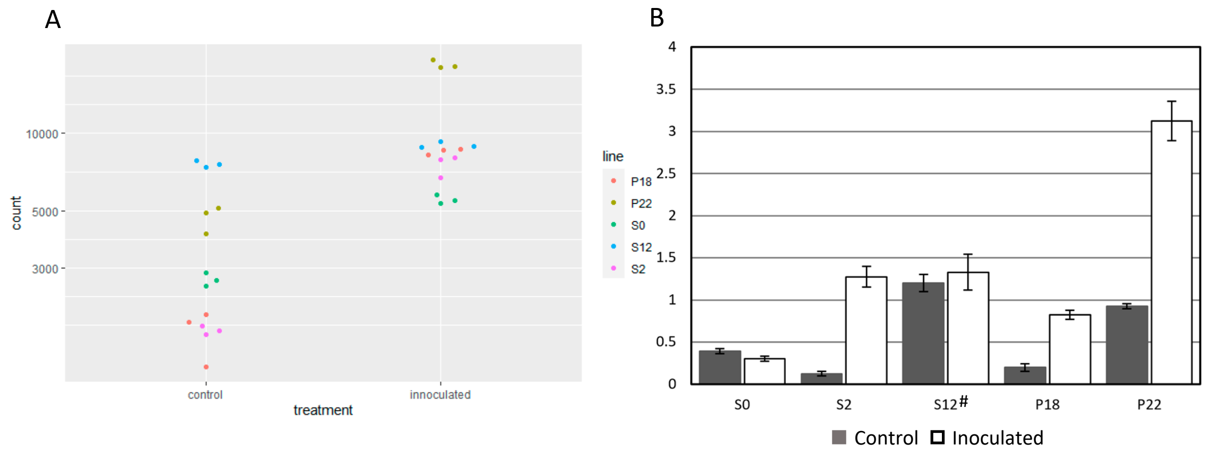

Pathogenesis-related_protein_PRB1-2_HORVU7Hr1G033530 (PR1B-2_ HORVU7Hr1G033530

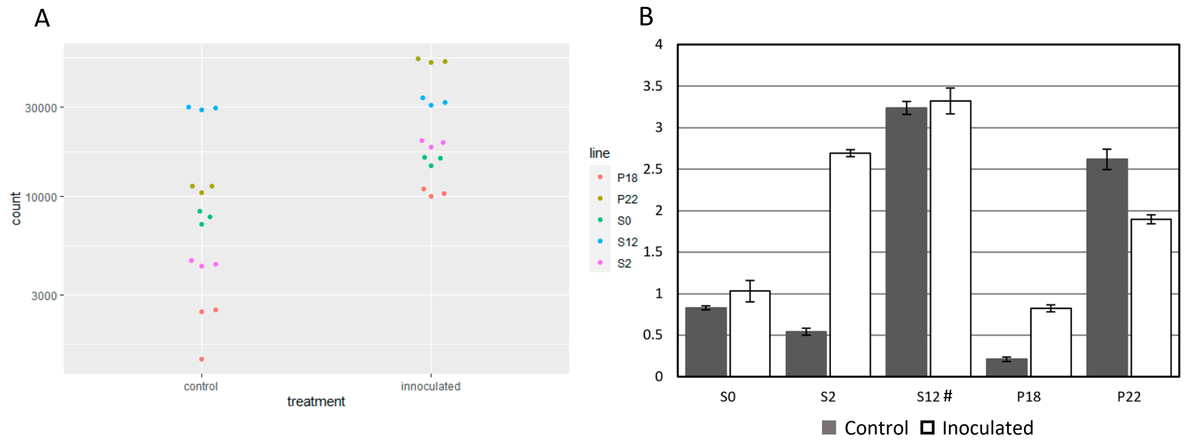

Supplement: Supplementary file 1 [file ijms-21-08769-s001.zip › CNV2-IJMS-SupplementaryFigures.pdf]
